# Caffeine in Opium-Coma

**Published:** 1860-09

**Authors:** Henry F. Campbell


*Caffeine in Opium-Coma.—The Second Case of the Injection of Caffeine, by the Rectum, in Extreme Narcotism of Opium.* By HENRY F. CAMPBELL.

In the May number of the *Southern Medical and Surgical Journal*, of the present year, we reported the particulars of a case of Opium-Coma, of a very grave character, in which *twenty grains of Caffeine*, injected into the rectum, produced the most surprising and satisfactory results. At the close of that former paper, we expressed the wish that some member of the Profession would repeat the treatment applied by us in that case, and either confirm or disprove our confidence in the remedy. The various medical journals of the country have commented upon the paper, and have generally approved the rationality of the measure, but, as yet, we have not been gratified by observing the report of any second trial of Caffeine under the circumstances, or any additional evidence in support of our favorable conviction in regard to the antidote. A case which occurred to us on the 10th of July instant, affords us the privilege of being able to report the second case of the application of Caffeine for Opium-Coma. Although the following case was not attended by the same happy eesults as that reported in our May number, we think that the details of the phenomena, so far from weakening our confidence in the remedy, will go far to confirm it.

July 10th, 1860, 3½ o'clock, P. M., called in haste to the U. S. Hotel, in this city, to visit a gentleman, said to have been found in a dying condition in one of the rooms. The patient was Mr. Moses Pike, a Jew, aged about 28 years, ot good constitution apparently, and well developed corporeally. On entering the room, we found him in the following condition: He was entirely unconscious; face of a dark purple hue; hands and feet also purple from congestion; nails on fingers and toes of an indigo color. There were also patches of venous congestion, presenting a darkened hue all over the surface. His respiration was fearfully slow when counted, *not quite four to the minute.* The attendants were slapping and shaking him each time between the inspirations, to excite him to breathe. His respiration seemed greatly obstructed by the accumulation of mucus. Pulse very feeble, and about 100 per minute. The muscular system was completely relaxed, so that his head would fall about by its own weight, and his arms and legs obeyed only the influence of gravity.

Immediately on our arrival, a paper was found, on which the unfortunate man had recorded the fact that he had taken laudanum at 12 o'clock the night previous, with the intention of self-destruction. Two empty vials, labelled laudanum, one of 2 ounce capacity, the other of 1 ounce, was found on the table. One of these vials had the neck knocked off, apparently with the view of opening it hastily—and some of the laudanum had escaped so as to leave a stain upon the label. It is probable, therefore, that the entire three ounces had not been taken. Once or twice during the morning, the servant stated, that he had approached and tried the door with the view of entering, but had desisted when he heard the occupant snoring deeply, as he did not wish to disturb him. Somewhat after 3 o'clock, P. M., the servant became alarmed and looked into the room through the transom-light from the chair, and observing his condition, called for assistance.

From the above circumstances, as well as from the written statement of the patient, it was highly probable that near 3

ounces of laudanum had been in his system nearly fifteen hours —that so large an amount had not produced death in so long a time, is truly unaccountable.

The condition of the patient, the necessity of constantly provoking respiration, and also the little probability that any laudanum yet remained in the stomach, caused us to abandon the idea of using the stomach pump. Emetics of course were out of the question, and we at once resorted to the application of ice to the scalp, and pouring ice-water, from a distance, upon the head, while we sent for a drachm of Caffeine, and a small syringe. As soon as these arrived, we poured out in the palm of the hand what we supposed to be about twenty grains of Caffeine, dissolved it in two ounces of cold water, and introduced it into the rectum by means of the syringe. The syringe being small, three applications were made at short intervals. The whole of the alkaloid was not dissolved. By an estimate made subsequently, calculating what had been lost, the patient had taken near *tweuty-five grains of Caffeine* in the three applications.

The Caffeine was administered at twenty minutes before four o'clock, at which time, as we have said, the respiration of the patient was *scarcely four to the minute*, and constant efforts were necessary, in the way of slapping and shaking to provoke him to inspire. At gfteen minutes after four, (35 minutes after the injection) his respiration was found to be effected with less effort and more regularly—and, on counting it by the watch, it numbered eight to the minute. The skin, even now, began to present less of the cerulean tint. In one hour after, the respiration had risen to twelve, and shortly rose to sixteen to the minute, when the skin was nearly of the natural hue, though the nails on both hands and feet remained still of a purplish cast.

Slight spasmodic movements in the fingers were now observed, and also some occasional subsultus in the muscles of the forearm —the under lip, which before was hanging, now became elevated ond slightly compressed against the teeth. When the hand of the patient was held, and an attempt made to extend the arm at the elbow, decided muscular resistance was observed. The lid of the left eye was also observed to be raised and let down rapidly once or twice.

The pulse hed now become full and somewhat resisting, and the action of the heart, as observed at the chest, tumultuous.—On being raised, the patient, once, made a noise slightly resembling a groan, but, from the beginning to the end, he did not once manifest the least consciousness.

For a short time after the improvement in respiration began, the mucous rale seemed somewhat to diminish, and his breathing, were it not for a certain jerking, resembled very nearly a man in deep, healthy sleep. The rale now, however, (½ past 7 o'clock) became more and more obstructive, the gurgling reaching up into the throat and threatening momentarily to strangle the patient. It was now plain that he could not survive, and on turning him upon the right side, a bloody mucus bubbled out of the nostrils. The number of the respirations was at this time twenty to the minute, when counted by the watch. The entire surface of the body was intensely hot, tnd remained so to the time of the patient's death, which took place at 15 minutes before 9 o'clock, P. M. He seemed to die from the accumulation of the bloody mucus, in the bronchial tubes and larynx. During the whole time, from the first moment of our seeing him till the time of his death, the application of ice was made constantly to the head of the patient, and also mustard plasters were applied to the spine and to the extremities.

A superficial glance at the foregoing case might, perhaps, impress the reader with the conviction that the confidence which we expressed, in our former report, in Caffeine as an antidote in Opium-Coma, was somewhat hasty and misplaced. A more deliberate consideration, however, will remove such an impression. When we reflect on the amount of the opium taken, the length of time during which the patient had been left to its toxic influence, and the destructive ravages which had been made during that time, we certainly, on the other hand, must feel great surprise at the amount of modification the Caffeine was seen to produce under such disadvantageous circumstances. The respiration, in a space of time, less than one hour, was raised from four to sixteen in the minute. The color of the skin, under its influence, was changed from an almost indigo hue, to that of the natural complexion, and the muscular relaxation was replaced by a fair degree of tonicity accompanied by occasional twitchings. The mode of death, too, was not such as is seen in the demise from the unmodified effects of opium, when the respiration becomes gradually slower till it ceases altogether, but at the time of our patient's death, his respiration numbered twenty per minute, and he died apparently *drowned* by the accumulation of the viscid mucus in the air-passages, doubtless the result of the long-enduring pulmonary congestion occurring previous to the administration of the Caffeine.

In conclusion, we feel confident in saying that we feel greatly encouraged by the developments of this second case, and shall use the remedy hereafter, with even more confidence than before. We again express the hope that some of our professional brethren will add their published testimony to ours so as to establish the true amount of value that should be attached to Caffeine as an antidote in Opium-Coma.

We intend shortly reporting the results of experiments, with the two drugs, Opium and Caffeine, as made by us, on the lower animals.*—*Southern Medical & Surgical Journal.*

*In the above case, at no time, did we find it necessary to resort to artificial respiration. The patient could always be incited to inspire naturally by shaking or pushing against his shoulder. The mode of artificial respiration described in our own report of the former case, has been supposed by some (our friend, Dr. E. Bland, of Edgefield) to be identical with that described by Dr. Sylvester, and reported in Braithwaite's Retrospect for 1859. We had not then seen Dr. Sylvester's method; we now admit that mode of procedure is nearly similar in both, but his involed the horizontal position, while ours presented the advantage of being applicable in the sitting posture, where the weight of the patient's body was made subservient in effecting the vacuum to cause the ingress of air into the lungs. As we have heretofore promised, we will hererfter give a full description of both methods, when the reader will be able to recognize the peculiarities of each.

